# Complete Genome Sequence of Ochrobactrum haematophilum FI11154, Isolated from Kunu-Zaki, a Nigerian Millet-Based Fermented Food

**DOI:** 10.1128/genomeA.00428-18

**Published:** 2018-05-17

**Authors:** Maria Diaz, Udo Wegmann, Nwanneka Akinyemi, Folarin A. Oguntoyinbo, Lizbeth Sayavedra, Melinda J. Mayer, Arjan Narbad

**Affiliations:** a Food and Health Institute Strategic Programme, Quadram Institute Bioscience, Norwich Research Park, Norwich, United Kingdom; b Gut Health and Food Safety Institute Strategic Programme, Quadram Institute Bioscience, Norwich Research Park, Norwich, United Kingdom; c Department of Microbiology, Faculty of Science, University of Lagos, Lagos, Nigeria

## Abstract

Ochrobactrum haematophilum FI11154 was isolated from kunu-zaki, a Nigerian traditional fermented millet-based food. Here, we present the first complete genome sequence of this species. The genome consists of five replicons and contains genes related to iron uptake and phosphatase activities.

## GENOME ANNOUNCEMENT

Here, we report the first complete genome sequence of Ochrobactrum haematophilum, a Gram-negative, nonmotile, rod-shaped, oxidase-positive bacterium from the Alphaproteobacteria class. This species was first isolated from human blood (1) but is frequently associated with plants (2) and the rhizosphere (3, 4). O. haematophilum has been reported to promote plant growth through the solubilization of phosphates and the production of siderophores (3, 4) and to produce fungicidal substances (4).

O. haematophilum FI11154 was isolated from kunu-zaki, a traditional Nigerian fermented food produced through the spontaneous fermentation of millet. Genomic DNA was extracted from a pure culture grown in Luria broth (5) using the cetyltrimethylammonium bromide-based extraction protocol (6), including RNase treatment. The genome was sequenced using the Pacific Biosciences (PacBio) RS II sequencing platform (Centre for Genomic Research, University of Liverpool, Liverpool, UK). *De novo* assembly of the read sequences was performed using the Canu 1.6 assembler (7), resulting in five contigs. PCR confirmed that the contigs correspond to five circular molecules. Annotation was performed using the Rapid Annotations using Subsystems Technology server version 2.0 (8).

Based on the definition of Harrison et al. (9), the genome of O. haematophilum FI11154 comprises one chromosome of 2,602,474 bp, with an average GC content of 57.51%, one chromid of 1,396,538 bp, with an average GC content of 57.5%, one megaplasmid of 1,035,251 bp, with an average GC content of 55.69%, and two plasmids of 266,969 and 181,071 bp with average GC contents of 55.78 and 55.59%, respectively. A total of 5,539 protein-coding sequences were identified, along with with 62 tRNA genes and 12 rRNA operons. Species identification was carried out by comparison of the 16S rRNA gene using the Seqmatch tool from the Ribosomal Database Project (10).

Several genes and gene clusters related to the uptake of iron were identified in the genome. Among them are the gene cluster *efeUOB,* putatively encoding an elemental ferrous iron transporter (11), the *fepC*, *fepG, fepD*, and *fepB* genes, putatively encoding a ferric enterobactin transport system, a gene cluster putatively encoding the vitamin B_12_ transport system BtuCDF (12), and several genes putatively encoding TonB-dependent transporters, which bind and transport ferric chelates, as well as vitamin B_12_, nickel complexes, and carbohydrates (13). The capacity of this microorganism to chelate iron can promote plant growth (3, 4); however, it could be detrimental to the nutritional value of the fermented foods from which it was isolated. The presence of two genes encoding putative exopolyphosphatases could be related to the phosphate solubilization activity described for this species (2, 4). Interestingly, a gene expected to encode an antilisterial bacteriocin (linocin M18 [14]) was predicted using antiSMASH 3.0 (15). The complete genome sequence of O. haematophilum FI11154 will contribute to a better understanding of the microbial diversity and dynamics of cereal-based fermented foods.

### Accession number(s).

The genome sequence has been deposited at the European Nucleotide Archive under the accession no. OOFM01000000.
